# Diagnostic accuracy of Bellavere’s score in cardiac autonomic neuropathy among chronic kidney disease patients: a study of prevalence and dialysis impact

**DOI:** 10.3389/fmed.2024.1514214

**Published:** 2025-01-10

**Authors:** Saket Satyasham Toshniwal, Sunil Kumar, Sourya Acharya, Abhishek Ghali, Sarang Raut, Vinit Deolikar, Harshitha Reddy, Palash Kotak, Anil Wanjari, Shilpa Bawankule

**Affiliations:** Department of General Medicine, Jawaharlal Nehru Medical College, Datta Meghe Institute of Higher Education and Research (DMIHER), Wardha, India

**Keywords:** Bellavere’s score, cardiac autonomic neuropathy, chronic kidney disease (CKD), dialysis, end stage renal disease, heart rate variability, Valsalva ratio, blood pressure response

## Abstract

**Background:**

Cardiac autonomic neuropathy (CAN) is a significant complication in chronic kidney disease (CKD), leading to increased morbidity and mortality. Early detection is essential for managing CKD patients effectively, especially those on hemodialysis. This study evaluated the prevalence CAN in CKD and diagnostic accuracy of Bellavere’s Score in predicting CAN in CKD patients, including those undergoing hemodialysis.

**Methods:**

This prospective observational study included 200 CKD patients. Cardiac autonomic neuropathy was assessed using Bellavere’s Score, calculated through a series of autonomic function tests including heart rate variability and blood pressure responses. Bellavere’s Score was measured pre-and post-dialysis in hemodialysis patients. The diagnostic performance of the score was evaluated using receiver operating characteristic (ROC) curves to determine sensitivity, specificity, and likelihood ratios.

**Results:**

Among the patients, 60% were diagnosed with CAN, with 35% having early CAN and 24% severe CAN. Bellavere’s Score showed high diagnostic accuracy across CKD stages, with sensitivity ranging from 75 to 89.29% and specificity from 69.09 to 96%. In CKD stage III patients, the sensitivity was 78.57% and specificity 91.4%. In stage V, sensitivity increased to 89.29%, though specificity dropped to 69.09%. For hemodialysis patients, Bellavere’s Score exhibited a sensitivity of 79.78% and specificity of 79.28%. The prevalence of CAN decreased significantly from 79.8% pre-dialysis to 64% post-dialysis (*p* < 0.01).

**Conclusion:**

Bellavere’s Score provides a reliable and non-invasive approach for diagnosing CAN in CKD patients, with strong diagnostic performance across different disease stages and in hemodialysis. Larger studies are warranted to further validate its utility.

## Introduction

Cardiac autonomic neuropathy (CAN) is a serious and often underdiagnosed complication in patients with chronic kidney disease (CKD), significantly contributing to cardiovascular morbidity and mortality. Patients with CKD predispose to a range of cardiovascular disorders, with CAN being one of the most debilitating due to its silent progression and association with poor clinical outcomes (1). Early detection of CAN is crucial for initiating timely interventions that may prevent life-threatening cardiac events. However, reliable and accessible diagnostic tools for CAN are limited, posing challenges to its routine clinical identification (2). Traditionally, the diagnosis of CAN has relied on a series of autonomic function tests, including heart rate variability (HRV) analysis, which require specialized equipment and expertise. The asymptomatic nature of early-stage CAN and overlapping symptoms with other CKD-related complications further make its detection more challenging. Studies suggest that up to 88% of patients with advanced CKD may exhibit autonomic dysfunction, yet understanding of CAN progression across CKD stages remains limited (3, 4).

Recent advancements in diagnostic tools have introduced promising alternatives for CAN assessment. Among these, Bellavere’s Score has emerged as a promising tool and it is a simple, non-invasive scoring system based on clinical parameters such as heart rate response to deep breathing, the Valsalva maneuver, and blood pressure changes (5–8). Furthermore, unlike Framingham Risk Score and QRISK, which primarily assess cardiovascular risk based on population-level epidemiological data, this score system focuses on direct measures of autonomic function, providing a more specific and practical approach to diagnosing CAN. Its non-invasive nature and reliance on simple clinical parameters make it superior for early detection and risk stratification in CKD patients with autonomic dysfunction. Originally developed and validated in diabetic populations, this tool has demonstrated significant utility in predicting CAN without requiring extensive resources (6, 7). However, its efficacy in CKD populations, where CAN prevalence is high, remains underexplored.

Cardiac autonomic neuropathy (CAN) is a significant but often underdiagnosed complication in chronic kidney disease (CKD). While several tools, such as those highlighted by Quarti-Trevano et al. (8), exist for assessing autonomic dysfunction, many require advanced equipment or specialized expertise, limiting their accessibility in routine clinical practice. This study aims to validate Bellavere’s Score as a simpler, non-invasive alternative for diagnosing CAN, particularly in CKD patients (8).

Therefore, the present study aimed to assess the prevalence of CAN across different CKD stages and evaluate the diagnostic accuracy of Bellavere’s Score for identifying CAN in CKD patients, providing insight into how autonomic dysfunction progresses with the advancement of kidney disease. By evaluating its predictive value in this specific population, we hope to establish Bellavere’s Score as a reliable tool for early detection and risk stratification of CAN in CKD patients. The findings of this study could facilitate improved screening practices and contribute to better cardiovascular outcomes in this vulnerable population.

## Methods

### Study design

This prospective observational cross-sectional study was conducted in the Department of Medicine at Acharya Vinoba Bhave Rural Hospital, a tertiary healthcare teaching center in Wardha district, Maharashtra, India. The study was conducted over a period of 2 years, from July 2022 to May 2024, and enrolled 200 participants diagnosed with chronic kidney disease (CKD) based on the criteria established by the National Kidney Foundation. The study was approved by the Institutional Ethics Committee of Datta Meghe Institute of Higher Education and Research (approval number ID-DMIMS (DU)/IEC/2022/1088), ensuring adherence to ethical guidelines, and written informed consent was obtained from all participants before enrollment.

### Study population

The study population included adult patients aged 18 years and above of either gender, who satisfied the diagnostic criteria for CKD. All participants provided informed consent for participation. The inclusion criteria ensured that only those with a confirmed diagnosis of CKD, as defined by the National Kidney Foundation - Kidney Disease Outcomes Quality Initiative (NKF-KDOQI), were included in the study. This included patients across various stages of CKD, enabling the evaluation of cardiac autonomic neuropathy (CAN) severity using Bellavere’s Score at different stages of renal impairment. Patients were excluded from the study if they were critically ill, required mechanical ventilation, or had a poor prognosis with death occurring within 24 h of admission. Patients who refused diagnostic investigations or declined to give consent for study procedures were also excluded. To ensure the study population was representative and to focus on non-acute cases, additional exclusion criteria were considered. These included patients with acute kidney injury (AKI), those with primary cardiovascular or autonomic nervous system diseases independent of CKD, and patients with coexisting chronic illnesses such as advanced liver disease or terminal cancer that could confound the assessment of CAN. Of the 220 participants initially recruited, 200 were included in the final analysis. A total of 20 participants (9.1%) were excluded due to various reasons, including failure to complete follow-up visits (*n* = 12), withdrawal of consent (*n* = 5), and mortality unrelated to study interventions (*n* = 3). These exclusions were documented, and attrition was accounted for during data analysis to minimize bias.

### Clinical examination and assessment

A comprehensive clinical assessment was conducted on all enrolled patients to evaluate their overall health and CKD status, starting with a detailed medical history that included details on pre-existing co-morbidities or conditions, medications, lifestyle factors, and duration of CKD. This was followed by a thorough physical examination, with a focus on identifying any cardiovascular complications, a common issue in patients with CKD. Key measurements such as blood pressure, heart rate, and body mass index (BMI) were recorded. Blood pressure variability was assessed according to recommendations of the American Heart Association (AHA) for measuring blood pressure, emphasizing standardized techniques, cuff calibration, and consistent measurement conditions. The assessment of Heart Rate Variability (HRV) was conducted following the guidelines set by the Task Force of the European Society of Cardiology and the North American Society of Pacing and Electrophysiology (1996). This guideline outlines standard methods for time-domain, frequency-domain, and non-linear analysis of HRV.

### Laboratory investigations

In addition to clinical evaluation, a series of laboratory tests were conducted for all participants. These included a complete blood count (CBC) to check for anemia or infection, kidney function tests (KFT) to evaluate serum creatinine and urea levels, serum albumin and total serum protein levels to assess nutritional status and kidney function, and an HbA1c test to monitor glycemic control in diabetic patients or screen for undiagnosed diabetes. Additionally, a lipid profile was performed to assess cardiovascular risk, serum electrolytes were tested to monitor sodium, potassium, and bicarbonate levels, and urine routine microscopy and urine microalbumin tests were conducted to detect proteinuria and other signs of kidney damage.

### Assessment of kidney function

Estimated glomerular filtration rate (eGFR) was calculated using the CKD-EPI formula, which provides an accurate assessment of kidney function. Staging of CKD was done according to the National Kidney Foundation-Kidney Disease Outcomes Quality Initiative (NKF-KDOQI) guidelines based on eGFR, as outlined in Table 1. Patients were classified into different CKD stages (1–5) based on their eGFR values.

**Table 1:** Staging of CKD according to NKF-KDOQI guidelines.

Stages	Groups	eGFR
Stage 1	GFR >90	111 ± 26.2
Stage 2	GFR 60–90	77 ± 23.8
Stage 3	GFR 30–59	39 ± 9.0
Stage 4	GFR 15–29	21 ± 6.0
Stage 5	GFR < 15	13 ± 4.5

### Cardiac autonomic neuropathy (CAN) assessment

To assess CAN, the Cardiac Autonomic Neuropathy System Analyser (CANS504) was used to calculate Bellavere’s Score. This score integrates several autonomic function tests, including heart rate and blood pressure responses, to evaluate the severity of CAN. The Bellavere’s Score ranged from 0 to 10, with a score of 0–1 indicating no CAN, 2–4 suggesting early CAN, and 5–10 indicating severe CAN. Specific tests to calculate Bellavere’s Score included measuring systolic blood pressure response to standing, diastolic blood pressure response to isometric exercise, heart rate variability during deep breathing, the Valsalva ratio, and the 30:15 ratio. Each test contributed to the final score, providing a detailed evaluation of the patient’s autonomic function (Table 2). Bellavere’s Score was measured post-dialysis in patients on hemodialysis and non-hemodialysis.

**Table 2:** Components of Bellavere’s score with its interpretation (6).

Components	Score		
	0	1	2
	Normal	Borderline	Abnormal
Heart rate variability	>15	10–15	<10
Valsalva ratio	≥1.21	1.11–1.20	≤1.10
30:15 ratio	≥1.04	1.01–1.03	≤1.0
BP response to standing	≥10	11–29	≥30
BP response to hand grip	≥16	11–15	≤10

The landmark studies by Ewing et al. (9–11) pioneered the development and validation of key methods for assessing variables in cardiac autonomic neuropathy, including systolic blood pressure response to standing, diastolic blood pressure response to isometric exercise, and heart rate variability measures such as the 30:15 ratio and Valsalva ratio as explained below in detail (9–11).

#### Systolic blood pressure response to standing

Systolic BP was measured while the patient was supine and again 2 min after standing. A drop of <10 mm Hg was considered normal, 10–29 mm Hg borderline, and ≥ 30 mm Hg with symptoms was considered abnormal (9).

#### Diastolic blood pressure response to isometric exercise

The subject squeezes a handgrip dynamometer to establish a maximum. Grip was then squeezed at 30% maximum for 5 min. A normal response for diastolic blood pressure was a rise of >16 mm Hg in the opposite arm (9, 10).

#### Heart rate variability (HRV) with deep breathing

Deep breathing at six breaths a minute was the most convenient technique that was reproducible. In this test, the subject was asked to breathe deeply at six breaths per minute, i.e., 5 s in and 5 s out for 1 min. The ECG was recorded throughout the period of deep breathing with a marker which was used to indicate the onset of each inspiration and expiration. The maximum and the minimal R-R intervals during each breathing cycle are measured by using a ruler and these are converted to beats per minute (9, 11).

The results of this test are expressed as the mean of the difference between the maximum and minimum heart rates for the six measured cycles in beats per minute. A value of ≤10 beats per minute was considered as abnormal reading with the patient at rest and supine, heart rate was monitored by ECG while the patient breathes in and out at 6 breaths/min, paced by a metronome or similar device (9, 11).

A difference in heart rate of >15 bpm was normal and < 10 bpm was abnormal. The lowest normal value for the expiration-inspiration ratio of the R-R interval was 1.17 in patients aged 20–24; this value decreases with age (9, 11).

#### Valsalva ratio procedure

The subject was asked to exhale into the mouthpiece which was connected to a mercury manometer, while holding it at a pressure of 40 mmHg for 15 s. During this maneuver and 45 s subsequent to this, the ECG was recorded and the valsalva ratio was calculated, which was the ratio between the maximal R-R interval (after the release of the strain) and the minimal R-R interval (during the strain). A ratio of ≤1.10 was considered as abnormal. The subject forcibly exhales into the mouthpiece of a manometer to 40 mm Hg for 15 s during ECG monitoring. Healthy subjects develop tachycardia and peripheral vasoconstriction during strain and an overshoot bradycardia and rise in blood pressure with release. The normal ratio of longest to shortest R-R was >1.2. The heart rate response to postural change (30:15 ratio) (9–11).

#### 30:15 ratio

The subject was made to lie quietly on a couch while the heart rate was continuously monitored on an electrocardiograph. The subject was then asked to stand unaided and the point at the starting to stand was marked on the electrocardiograph. The shortest R-R interval at or around the 15th beat was measured, while the longest R-R interval at around the 30th beat after standing was measured. A ratio of ≤1 was considered as abnormal (9, 11).

During continuous ECG monitoring, the R-R interval was measured at beats 15 and 30 after standing. Typically, a tachycardia was followed by reflex bradycardia. The 30:15 ratio should be >1.03 (9, 11).

Autonomic functions were assessed using standardised techniques including valsalva maneuver, deep breathing and heart rate variability. Prior studies, such as Suzuki et al. (12) have demonstrated the utility of baroreflex latency analysis during the valsalva maneuver for assessing autonomic neuropathy, reinforcing the importance of Valsalva testing in autonomic evaluations and emphasizing on broader relevance of valsalva maneuver in autonomic assessments (12).

However, our study primarily focuses on valsalva ratio calculations to demonstrate autonomic responses consistent with established protocols by (9, 11).

### Statistical analysis

The Shapiro-Wilk test was used to assess data distribution. Non-parametric tests (Mann-Whitney U or Kruskal-Wallis) were applied if normality was not met, while parametric tests (t-test or ANOVA) were used for normally distributed data. Significance was set at *p* < 0.05.

### Calculation of sensitivity and specificity

Sensitivity and specificity were calculated based on the results of Bellavere’s Score in diagnosing cardiac autonomic neuropathy (CAN) using the following definitions: Sensitivity was calculated as the proportion of true positives (patients diagnosed with CAN by Bellavere’s Score and confirmed by clinical criteria) out of all actual positives (total patients confirmed to have CAN by clinical criteria).

Specificity was calculated as the proportion of true negatives (patients not diagnosed with CAN by Bellavere’s Score and confirmed to not have CAN by clinical criteria) out of all actual negatives (total patients confirmed to not have CAN by clinical criteria).

The diagnostic accuracy metrics were determined for each CKD stage and for the total study population. The reference standard for confirming CAN was based on clinical criteria derived from autonomic function tests.

#### Validation using ROC analysis

Receiver operating characteristic (ROC) curve analysis was conducted to evaluate the overall diagnostic performance of Bellavere’s Score. The area under the curve (AUC) was used to assess the score’s ability to distinguish between patients with and without CAN across different CKD stages. Graphical representations of the results were used whenever it was judged necessary. For the majority of the study, SPSS Version 26.0 was used, while Microsoft Excel 2021 was utilised for the graphical depiction.

## Results

### Baseline patient characteristics

Out of the 220 participants initially enrolled, 20 were excluded during the study period due to loss of follow-up, withdrawal of consent and unrelated mortality. The remaining total of 200 participants whose data were analyzed, as summarized in Table 3. In the study, the mean age of participants was 53.56 years, with 38.5% of patients aged over 60 years. There was a clear male predominance, with 70% of the cases being male compared to 30% female. Hypertension was identified as the most common underlying cause of chronic kidney disease (CKD), affecting 43% of patients, followed by comorbid diabetes and hypertension in 13% of cases. Other etiologies included obstructive uropathy (6.5%), diabetic nephropathy, and nephritic syndrome with hypertension (2% each). Approximately 30% of patients had an unspecified etiology. The distribution of CKD stages showed that 0.5, 1.5, and 7% of patients were in CKD stages I, II, and III, respectively, while the majority were in stages IV (23.5%) and V (67.5%), with 44.5% requiring dialysis. Out of the total 200 cases, 0.5, 1.5 and 7% were in CKD stage I, II and III, while 23.5 and 67.5% were in stage IV or stage V respectively, while dialysis was required in 44.5% cases. The details of baseline characteristics are summarized in Table 3.

**Table 3:** Baseline characteristics of study participants including age, gender, and etiologies.

Parameters	*N* = 200	%
Age group (years)		
18–40	40	20.0%
41–60	83	41.5%
>60	77	38.5%
Gender		
Female	60	30.0%
Male	140	70.0%
Etiology		
Hypertension	86	43.0%
Unspecified (CKD-U)	60	30.0%
Hypertension + Diabetes	28	14.0%
Obstructive uropathy	13	6.5%
Diabetes	4	2.0%
Others	8	4.5%

### Association of cardiac autonomic neuropathy with CKD stages

In our study, the association of CAN with various stages of CKD is summarized in Table 4. Among patients in CKD stages I and II, none exhibited CAN (*p* = 0.4 and *p* = 0.063, respectively; Fisher’s exact test, not significant). However, in CKD stage III, CAN was observed in 28.6% of patients, with the association reaching statistical significance (*p* = 0.021; Fisher’s exact test). In CKD stage IV, 57.4% of patients had CAN, but the association was not statistically significant (*p* = 0.735; Chi-square test). The prevalence of CAN was highest in CKD stage V, affecting 65.9% of patients, with a statistically significant association (*p* = 0.020; Chi-square test). Overall, CAN was present in 60% of the total study population, and the association between CKD stage and CAN was significant (*p* = 0.0077; Chi-square test). This suggests that the prevalence of CAN increases with the progression of CKD, particularly becoming more prominent and statistically significant in the later stages of the disease.

**Table 4:** Association of cardiac autonomic neuropathy (CAN) with CKD stages.

CKD stage	CAN		*p*-value	Total
	No	Yes		
I	1	0	0.4 Fisher’s exact test, Not significant	1
	100.0%	0.0%	–	100.0%
II	3	0	0.063 Fisher’s exact test, Not significance	3
	100.0%	0.0%	–	100.0%
III	10	4	0.021 Fisher’s exact test, significant	14
	71.4%	28.6%	–	100.0%
IV	20	27	0.735 Chi-square test, not significant	47
	42.6%	57.4%	–	100.0%
V	46	89	0.020 Chi-square test, significant	135
	34.1%	65.9%	–	100.0%
Total	80	120	0.0077	200
	40.0%	60.0%	–	100.0%
	Overall P value – 0.0077 (≈0.01) using Chi-square test			

### Association of cardiac autonomic neuropathy with dialysis requirement

The study revealed a significant association between the requirement of dialysis and the prevalence of cardiac autonomic neuropathy (CAN). Among patients not requiring dialysis, 44.1% had CAN, while a significantly higher prevalence of 79.8% was observed in those requiring dialysis (*p* = 0.0018). Additionally, an improvement in Bellavere’s score post-dialysis was noted in 75.3% of cases, while 24.7% showed no change. A comparison of pre-and post-dialysis CAN prevalence showed a reduction from 79.8 to 64% post-dialysis, indicating a significant improvement (*p* = 0.01), as detailed in Table 5.

**Table 5:** Comparison of pre-and post-dialysis cardiac autonomic neuropathy (CAN) prevalence.

CAN*	Pre-dialysis		Post-dialysis		*P*-Value
Yes	71	79.8%	57	64.0%	0.01
No	18	20.2%	32	36.0%	–
Total	89	100.0%	89	100.0%	–
*P*-value	0.0018		0.0469		

### Diagnostic accuracy of Bellavere’s score in detecting cardiac autonomic neuropathy across CKD stages

The Bellavere’s Score demonstrated robust diagnostic accuracy in detecting cardiac autonomic neuropathy (CAN) across various stages of chronic kidney disease (CKD). Sensitivity ranged from 75% in early-stage CKD (I and II) to 89.29% in stage V CKD, while specificity varied from 69.09 to 96% across stages. The diagnostic performance of the Bellavere’s Score was further confirmed through the ROC curve in patients undergoing hemodialysis. The score achieved a sensitivity of 79.78%, specificity of 79.28%, a positive likelihood ratio of 3.85, and a negative likelihood ratio of 0.26, highlighting its reliability in diagnosing CAN across different CKD stages (Table 6).

**Table 6:** Diagnostic accuracy of Bellavere’s score in various stages of CKD.

	Stage I and II CKD		Stage III CKD		Stage IV CKD		Stage V CKD	
Statistic and stages	Value	95% CI	Value	95% CI	Value	95% CI	Value	95% CI
Sensitivity	75.00%	19.41 to 99.37%	78.57%	49.20 to 95.34%	78.72%	64.34 to 89.30%	89.29%	72.25 to 86.39%
Specificity	96.00%	92.27 to 98.26%	91.40%	86.41 to 95.00%	79.74%	72.49 to 85.80%	69.09%	68.23 to 88.90%
Positive likelihood ratio	18.75	7.75 to 45.38	9.13	5.31 to 15.71	3.89	2.74 to 5.50	3.89	2.44 to 6.55
Negative likelihood ratio	0.26	0.05 to 1.42	0.23	0.09 to 0.64	0.27	0.15 to 0.47	0.27.	0.17 to 0.36
Disease prevalence (*)	1.96%	0.54 to 4.94%	7.00%	3.88 to 11.47%	23.50%	17.81 to 30.00%	67.50%	60.53 to 73.94%
Positive predictive value (*)	27.27%	13.42 to 47.58%	40.74%	28.55 to 54.18%	54.41%	45.74 to 62.82%	89.26%	83.53 to 93.15%
Negative predictive value (*)	99.48%	97.23 to 99.90%	98.27%	95.40 to 99.36%	92.42%	87.50 to 95.51%	65.82%	57.37 to 73.38%
Accuracy (*)	95.59%	91.79 to 97.96%	90.50%	85.56 to 94.18%	79.50%	73.23 to 84.87%	80.00%	73.78 to 85.31%

### Diagnostic accuracy of Bellavere’s score for predicting cardiac autonomic neuropathy across CKD stages and in hemodialysis patients

**Figure 1:**
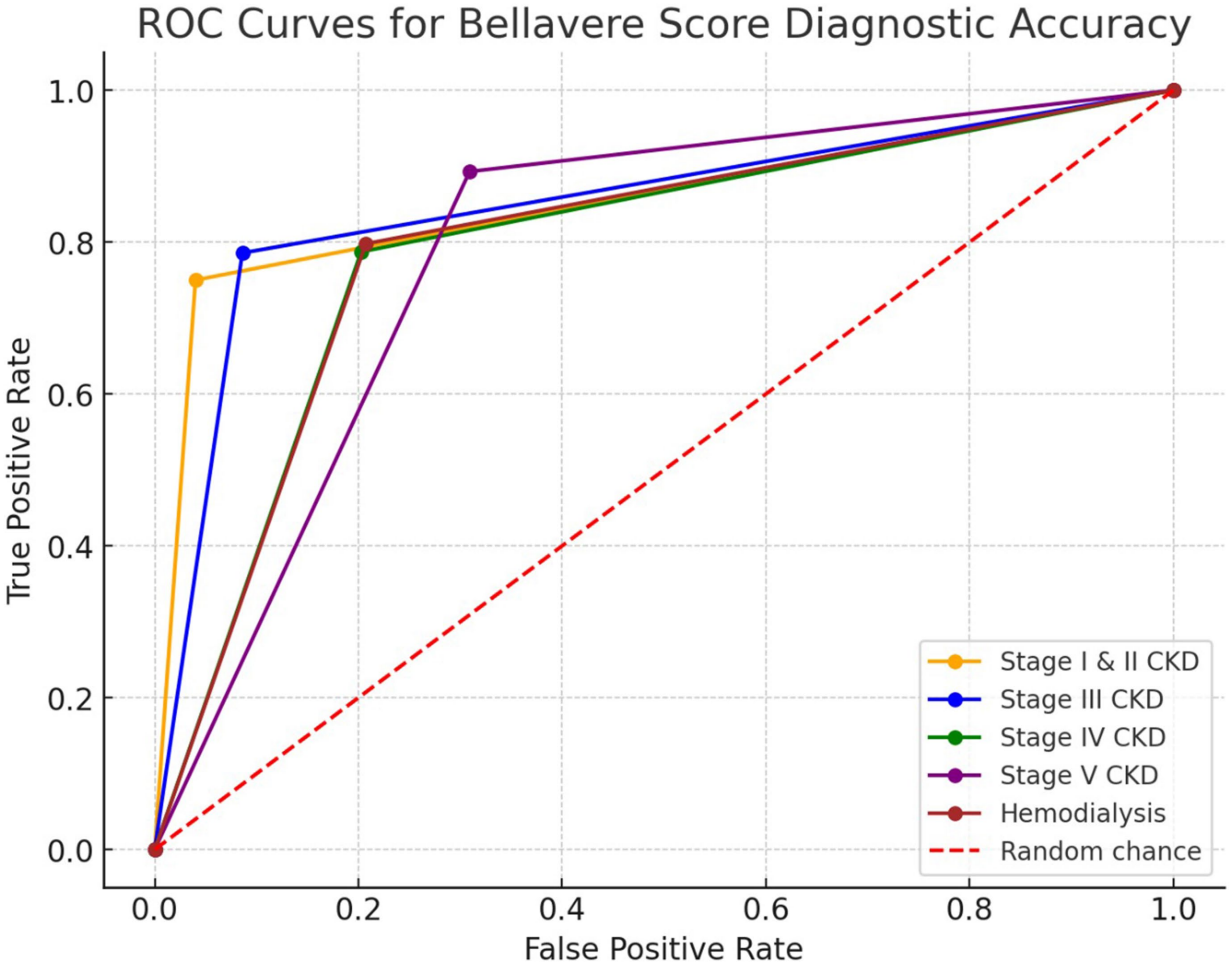
A ROC curve for diagnostic accuracy of Bellavere’s score for predicting CAN in different CKD stages in hemodialysis patients. The ROC curves depict the sensitivity and specificity of the Bellavere’s score in predicting CAN across CKD stages I–V, and in hemodialysis patients. Sensitivity and specificity vary across stages, with the highest sensitivity (89.29%) observed in stage V CKD, and the highest specificity (96.00%) in stages I and II. In hemodialysis patients, the score demonstrated a sensitivity of 79.78% and specificity of 79.28%, reflecting its overall diagnostic utility.

The diagnostic accuracy of the Bellavere’s Score in predicting CAN in different stages of CKD, as well as in patients undergoing hemodialysis, is illustrated in Figure 1. The ROC curve for stages I and II CKD shows a sensitivity of 75.00% and specificity of 96.00%, with a positive likelihood ratio of 18.75 and a negative likelihood ratio of 0.26. In stage III CKD, the sensitivity is 78.57% and specificity is 91.40%, with a positive likelihood ratio of 9.13 and a negative likelihood ratio of 0.23. For stage IV CKD, the sensitivity is 78.72% and specificity is 79.74%, with a positive likelihood ratio of 3.89 and a negative likelihood ratio of 0.27. In stage V CKD, the sensitivity increases to 89.29%, while specificity is 69.09%, with a positive likelihood ratio of 3.89 and a negative likelihood ratio of 0.27. Among patients on hemodialysis, the sensitivity is 79.78% and specificity is 79.28%, with a positive likelihood ratio of 3.85 and a negative likelihood ratio of 0.26.

## Discussion

In this study, we evaluated the diagnostic accuracy of the Bellavere’s Score in predicting CAN across various stages of CKD and in patients undergoing hemodialysis. Our findings indicate that the Bellavere’s Score is a valuable tool for identifying CAN, demonstrating significant sensitivity and specificity, particularly in advanced stages of CKD. The observed prevalence of CAN in our cohort aligns with existing literature, reinforcing the notion that autonomic dysfunction is a common complication in CKD patients. The notable variations in sensitivity and specificity across CKD stages highlight the need for tailored approaches in assessing and managing cardiac autonomic function in this vulnerable population. Herein, we further explore the implications of our findings in clinical practice, the potential mechanisms underlying the relationship between CKD and CAN, and the relevance of the Bellavere’s Score in enhancing patient outcomes.

We believe that the findings of this study may have clinical significance for several reasons. First, the study demonstrates the diagnostic accuracy of the Bellavere’s Score in predicting CAN across different stages of CKD, providing a reliable, non-invasive tool for early detection and monitoring of CAN in this patient population. Second, the high prevalence of CAN observed in later stages of CKD, particularly in patients requiring dialysis, emphasizes the need for routine screening and timely interventions to manage this complication. Third, the improvement in CAN prevalence post-dialysis suggests that dialysis may have a beneficial effect on autonomic function, supporting the use of this therapeutic approach not only for renal function management but also for cardiovascular risk reduction. Finally, the study highlights the association between uremic toxins and CAN severity, suggesting potential pathways for targeted therapies aimed at reducing uremic toxin levels, which could improve patient outcomes.

CKD is characterized by a sustained decline in glomerular filtration rate (<60 mL/min for 3 months or more) and histological evidence of nephron loss, posing a significant global public health challenge (13). Cardiovascular autonomic dysfunction represents a serious and often overlooked long-term complication in individuals with CKD (14, 15).

One of the significant findings in chronic kidney disease (CKD) is the impact of kidney innervation on the autonomic nervous system (ANS). The kidneys are richly innervated by sympathetic nerves, and in CKD, heightened renal sympathetic activity contributes to systemic autonomic dysfunction. This overactivation can lead to increased cardiovascular risk, progression of renal impairment, and a higher prevalence of cardiac autonomic neuropathy (CAN). The disruption of this neurohumoral balance, particularly in advanced CKD stages, underscores the intricate relationship between kidney function and ANS regulation. Incorporating this understanding into the present study, the high prevalence of CAN observed in advanced CKD stages could partly be attributed to enhanced renal sympathetic activity, which exacerbates autonomic dysfunction (8). Numerous studies have established that patients with CKD and concurrent cardiovascular autonomic dysfunction are at an increased risk of premature mortality (16–18).

Despite a proven reliability of cardiovascular reflex tests in the assessment of autonomic dysfunction, several caveats must be noted about their utilization in clinical practice. Ewing et al. (11) pioneered this field with a series of seminal studies conducted in 1974, 1981, and 1985, outlining a battery of standardized non-invasive tests, including heart rate variability (HRV) with deep breathing, the Valsalva maneuver, and the 30:15 ratio. These tests remain as quite effective for assessing autonomic dysfunction inspite of newly developed techniques that claim to replace these tests, the basic rationale of the technique lies in its reproducibility of the results and less complicated tests for detecting parasympathetic and sympathetic deficits. The Valsalva ratio, which is our major area of interest, has been shown, through Ewing’s work, to be a sensitive measure of autonomic dysfunction based on changes in heart rate during and following Valsalva maneuver (9–11).

However, Suzuki et al. (12) herein proposed a finer tuned method by examining the latency of tachycardia and bradycardia during the Valsalva maneuver to understand baroreflex. Their study emphasized the importance of baroreflex sensitivity measured as the latency of delayed bradycardia responses; their work presented a more complex view of parasympathetic dysfunction. This latency-based approach augments conventional autonomic tests, which can only look for imbalances in numbers, while this method targets dysfunctional changes that may not be recognizable at a ratio level (12).

As for the method we adopt in the present study, as mentioned above, we mainly apply the Valsalva ratio calculations and HRV analysis as suggested by the work of Ewing et al. (11). Nonetheless, the study of Suzuki indicates that there is a prospect of additional diagnostic value by using baroreflex latency. However, the simplicity and clinical utility of ratio-based assessments, as will be demonstrated by Ewing, remain a framework for widespread autonomic testing. Despite its clear benefit, the prospective part of the work, namely the analysis of latency, may need additional specialized equipment and software, which reduces the applicability of this information to everyday clinical activity (9–12).

Altogether, the researches of Ewing et al. (11) and Suzuki et al. (12) demonstrate the process of changes in the existing approaches to autonomic function testing. The results linking to protocols embedded on Valsalva ratios and HRV correspond to these historical and modern strategies as they demonstrate the benefits of traditional autonomic tests simultaneously affirming the proposals of the baroreflex advanced study for future works of the field (9–12).

In our study, the average age of participants was 53.56 years, with 38.5% being over 60 years of age. The demographic breakdown revealed a male predominance, with 60% of cases being male and 30% female. Research by Kaliya et al. (19) reported that 68% of CKD patients were in their fourth to eighth decades of life, also showing a slight male predominance. Pathak et al. (20) observed an average age of 69.1 years, with males comprising 56% of their study cohort. Similarly, Lahariya et al. (21) found that the majority of patients fell within the 51–60 age range, accounting for 23% of their total.

In our study, hypertension was the leading cause of CKD, accounting for 43% of cases. This was followed by comorbid diabetes and hypertension at 13%, obstructive uropathy at 6.5%, and both diabetic nephropathy and nephritic syndrome with hypertension at 2% each. Notably, 30% of cases had an undetermined cause. Similar findings have been reported nationally and globally. For instance, hypertension was identified as the cause in 26% of patients, diabetes in 22%, and glomerulonephritis, interstitial nephritis, or polycystic kidney disease in 27%, with other causes making up 26% (22–26). Additionally, Pathak et al. (20) noted that 45% of participants had diabetes, 38% had hypertension, and 8% had obstructive uropathy, while 9% had an unspecified etiology (20).

The results of this study indicate that a significant proportion of patients (67.5%) were in CKD stage V, with an additional 23.5% in stage IV, underscoring the advanced progression of chronic kidney disease in the cohort. Only a small percentage of patients were identified in earlier stages (CKD stages I–III), highlighting that CKD often remains undetected until it reaches more severe stages. This finding aligns with previous research by Kaliya et al. (19) and Lahariya et al. (21) where higher proportions of patients in advanced stages (III–V) were reported. The high rate of dialysis dependence in our study (44.5%) is consistent with the findings from Kaliya et al. (19) who reported 44% and Lahariya et al. (21) (dialysis required in 70% of stage V cases), indicating a critical need for renal replacement therapy in advanced CKD stages. However, the variation in the proportion of patients across different stages of CKD in these studies suggests potential differences in patient demographics, healthcare accessibility, or disease detection rates across regions.

In addition, the findings also suggest that 60% of CKD patients had CAN highlight the substantial burden of autonomic dysfunction in this population. The higher prevalence of CAN in advanced CKD stages (IV and V) compared to earlier stages (I and II) emphasizes the progressive nature of both CKD and its associated complications. The significant difference in mean age between patients with and without CAN (55.38 vs. 50.84 years, *p* = 0.023) suggests that older age may be a contributing factor to the development of CAN. However, the lack of a significant gender difference in prevalence (*p* = 0.34) implies that both men and women are equally susceptible to CAN in CKD. These findings align with previous research, such as Bokhari et al. (4) who reported an even higher prevalence of CAN (88%) in end-stage renal disease (ESRD) patients. The strong association between dialysis dependence and CAN (79.8% in dialysis-dependent patients vs. 46.1% in non-dialysis patients, *p* < 0.01) underscores the role of dialysis in either contributing to or unmasking autonomic dysfunction in CKD. Recovery in autonomic dysfunction after dialysis may be due to the rapid clearance of uremic toxins, correction of fluid and electrolyte imbalances, and improved baroreceptor sensitivity. Dialysis also reduces systemic inflammation and oxidative stress, key contributors to autonomic dysfunction. Additionally, normalization of cardiovascular hemodynamics further aids in the swift restoration of autonomic function, even in this chronic condition. The comparison with studies by Thapa and co-workers (27), Almakramy and his team (28), and Orlov and colleagues (29) further supports the link between CKD progression and the development of CAN.

Next, our study also highlights the potential role of protein-bound uremic toxins like indoxyl sulfate (IS) and p-cresyl sulfate (PCS) in contributing to CAN in ESRD, although direct evidence remains limited. The improvement in Bellavere’s Score observed in 75.3% of patients post-dialysis, alongside the significant reduction in CAN prevalence from 79.8 to 64% (*p* < 0.01), suggests that dialysis may mitigate CAN severity in many patients. This aligns with findings from Orihuela et al. (30), who demonstrated enhanced cardiac autonomic function with both hemodialysis and peritoneal dialysis. Additionally, Cheng et al. (31) showed that peritoneal dialysis reduces uremic toxin levels, further supporting its positive impact on CAN outcomes.

Sahin, Kayatas et al., 2006, validated Bellavere’s Score as a simple, non-invasive tool for diagnosing cardiac autonomic neuropathy, focusing on chronic kidney disease patients. Their work provided early evidence of its clinical utility, emphasized accessible bedside testing, and laid the groundwork for extending its use to broader populations, including CKD patients and its pioneering role in introducing accessible autonomic testing methods, which set the stage for further research in broader patient populations (32).

Our findings demonstrate that Bellavere’s Score is a robust diagnostic tool for CAN across CKD stages, with greater sensitivity and specificity compared to its initial validation in chronic kidney disease patients (32). Unlike prior studies, our work incorporates a larger sample size of 200 CKD patients, representing all CKD stages, including both dialysis and non-dialysis patients. This comprehensive approach not only highlights the diagnostic utility of Bellavere’s Score but also provides novel insights into stage-specific prevalence and the ameliorating effects of dialysis on CAN severity (32).

Finally, the Bellavere’s Score demonstrated strong diagnostic accuracy in detecting CAN at different stages of CKD, highlighting its utility as a reliable screening tool. With sensitivity ranging from 75 to 89.29% and specificity from 69.09 to 96%, it effectively identified patients at risk for CAN across various CKD stages. In hemodialysis patients, the score maintained a sensitivity of 79.78% and specificity of 79.28%, which reflects its robustness in this subgroup. A positive likelihood ratio of 3.85 indicates that patients with a positive Bellavere’s Score are nearly four times more likely to have CAN, while a negative likelihood ratio of 0.26 signifies that the test is also effective at ruling out CAN in those who test negative. This result underscores the score’s clinical value in managing CKD patients, particularly in identifying those who may benefit from early intervention for CAN.

Overall, the findings of the present study demonstrate that Bellavere’s Score effectively identifies autonomic dysfunction, with its predictive accuracy varying across CKD stages. Specifically, the score showed significant association with CAN prevalence in advanced CKD stages (Stages III and V), indicating that autonomic dysfunction worsens with disease progression. The high prevalence of CAN in Stages IV and V suggests that autonomic dysfunction is intricately linked to the progression of renal dysfunction, likely due to the cumulative effects of uremic toxins, chronic inflammation, and oxidative stress, which are hallmarks of CKD. Bellavere’s Score’s sensitivity to these changes underscores its potential as a practical, non-invasive tool for early detection and risk stratification of CAN, allowing timely interventions that could mitigate cardiovascular risks in this vulnerable population. Furthermore, the observed improvement in CAN prevalence post-dialysis suggests a potential role of dialysis in temporarily alleviating autonomic dysfunction, possibly through the removal of uremic toxins and correction of fluid and electrolyte imbalances. However, the persistence of autonomic dysfunction in a substantial proportion of patients post-dialysis indicates that CAN is a chronic condition requiring long-term management beyond dialysis.

Despite the valuable insights provided by this study, several limitations must be acknowledged. First, the cross-sectional nature of the study restricts the ability to establish causal relationships between CAN and CKD progression. Longitudinal studies would be necessary to better understand the temporal dynamics between these conditions. Second, the study was conducted at a single center, which may limit the generalizability of the findings to other populations with different demographic and clinical characteristics. Additionally, the sample size, although sufficient for detecting associations, may have limited the ability to analyze subgroups in more detail, particularly when examining the effects of dialysis on CAN severity. Finally, the use of the Bellavere’s Score, while effective, may not capture the full spectrum of CAN manifestations, as it was measured only once during the study. Repeated assessments of Bellavere’s Score would be necessary to analyze the long-term effects of dialysis on CAN. Additionally, incorporating other established gold standard diagnostic methods to compare and validate the diagnostic accuracy of Bellavere’s Score could provide a more comprehensive assessment of CAN. Further research is needed to validate these findings in larger, multicenter cohorts with stage specific sample size and to explore potential interventions for CAN in CKD patients.

## Conclusion

In conclusion, the findings underscore the utility of Bellavere’s Score as a reliable tool for diagnosing CAN across different CKD stages, particularly in patients requiring dialysis. These results emphasize the importance of early detection and proactive management of CAN in CKD patients to mitigate cardiovascular risks and improve overall outcomes. Future studies with larger sample sizes and longitudinal designs are warranted to further validate these findings and explore potential therapeutic strategies for CAN in CKD.

## Key learning points

### What is known?

Although several diagnostic tools for measuring autonomic dysfunction are available, many are limited by the need for advanced equipment or specialized expertise, making them less accessible for routine clinical use. This study validates Bellavere’s Score as a simpler, non-invasive, and clinically practical alternative for diagnosing CAN, particularly in CKD patients.

### This study adds

- Bellavere’s Score effectively diagnoses cardiac autonomic neuropathy in CKD patients, showing high predictive accuracy.
- This study confirms its utility for early detection and risk assessment of CAN in CKD.
- Key message: Use Bellavere’s Score to enhance early diagnosis and management of cardiac autonomic neuropathy in CKD.

### Potential impact

- Incorporating Bellavere’s Score into CKD assessments can enhance early detection of cardiac autonomic neuropathy.
- It may lead to personalized treatment plans and improved patient management.
- Policy adjustments could standardize its use, improving diagnostic accuracy and care in CKD patients.

## Data Availability

The original contributions presented in the study are included in the article/supplementary material, further inquiries can be directed to the corresponding author.
